# Evidence Summary for Nonpharmacological Management of Chemotherapy-Induced Nausea and Vomiting

**DOI:** 10.1155/2022/4741193

**Published:** 2022-11-23

**Authors:** Kexuan Li, Yuanyuan Cai, Shu Xie, Yingshuyi Zhou, Jiaqian Dong, Qingbo Zhu, Jiayu Zhang, Xiuyue Qiu

**Affiliations:** ^1^School of Nursing, Zhejiang Chinese Medical University, Hangzhou 310053, China; ^2^Ningbo No. 2 Hospital, Ningbo 315000, China

## Abstract

**Objective:**

To evaluate and summarize the best evidence for nonpharmacological management of chemotherapy induced nausea and vomiting (CINV). This article aims at serving as a reference for nursing staff to prevent and manage CINV.

**Methods:**

We systematically searched for evidence on CINV in databases such as Cnki and Wanfang, guide websites such as GIN and NZGG, and professional websites such as UICC and ACS. Only articles published between November 2012 and November 2021 were considered for inclusion in the summary. Two researchers evaluated the quality of the literature and extracted the data.

**Results:**

A total of 22 articles were included in this study, including 3 clinical decisions, 2 guidelines, 1 evidence summary, 2 recommended practices, 13 systematic reviews, and 1 expert consensus. Finally, 15 pieces of evidence were summarized from 3 perspectives: risk factor assessment, nursing process specification, and nonpharmacological interventions.

**Conclusion:**

Our research summarizes the best evidence on nonpharmacological management of CINV. During actual clinical application, it is necessary to fully consider the clinical situation, combine professional judgment with patients' wishes, follow the principle of individualization, analyze the obstacles and facilitating factors of the application of evidence, and prudently apply the evidence to the clinical practice.

## 1. Introduction

Chemotherapy induced nausea and vomiting (CINV) is the most common adverse reaction during chemotherapy in cancer patients [1]. Frequent and severe vomiting can elicit a loss of appetite, which leads to nutritional deficiency, decreased immunity, metabolic disorders, and additional adverse outcomes that affect the patient's quality of life and chemotherapy efficacy [2]. Based on its time of onset, CINV can be subdivided into acute, delayed, and expected nausea and vomiting according to onset time. Current antiemetic methods for chemotherapy can be pharmacological or nonpharmacological. Pharmacological antiemetic treatments include 5-HT3 receptor antagonists, NK-1 receptor antagonists, glucocorticoids, and other drugs. Although the occurrence of CINV has significantly improved in recent years, about 40% of patients still cannot effectively relieve CINV [3]. Nonpharmacological measures are typically used as adjunctive therapy to pharmacological antiemetics and have received increased attention because they are less risky, more affordable, safer, and more effective than pharmacological interventions [4]. Nursing staff are crucial to implementing nonpharmacological measures for CINV. They should improve awareness of and attention to these interventions and actively carry out these interventions in clinical practice [5]. Therefore, this study extracted and summarized the evidence on current CINV nonpharmacological management and provided references for the clinical practice of CINV nonpharmacological management to help medical staff make decisions more scientifically.

## 2. Materials and Methods

### 2.1. Question Identification

This study aims at identifying nonpharmacological measures that can effectively prevent and manage CINV. To obtain the best evidence, the PICOS principle is utilized to form the initial questions for evidence-based care. In the context of this study, the P (for “population”) refers to cancer patients age ≥ 18 years. The I represents “intervention” and refers to the various nonpharmacological measures that could prevent, manage, treat, or nurse CINV. The C stands for “comparison”, which is the current clinical nursing measure. The outcome, or O, refers to the outcome index for CINV, which includes physiological and psychological indicators. Lastly, S stands for “study design” and, in this study, refers to clinical decisions, guidelines, evidence summaries, recommended practices, systematic reviews, and expert consensus.

### 2.2. Retrieval Strategy

Using the “6S” evidence model, evidence retrieval time was from November 2012 to November 2021. The databases were UpToDate, BMJ, Zynx, DynaMed, Clinical key, Cochrane, Joanna Briggs Institute Library, EBSCO Nursing Reference Center, CINAHL, Web of Science, PubMed, Embase, ProQuest, CBM, Cnki, Wanfang, and VIP. Guidance website included WHO, Medlive, American Society of Clinical Oncology (ASCO), Scottish Intercollegiate Guidelines Network (SIGN), Institute for Clinical and Economic Review (ICER), New Zealand Guidelines Group (NZGG), National Institute for Health and Care Excellence (NICE), Guidelines International Network (GIN), and National Guideline Clearinghouse (NGC). Professional Society website included American College of Physicians (ACP), Union for International Cancer Control (UICC), American Cancer Society (ACS), and Registered Nurses Association of Ontario (RNAO). The Chinese search keywords were “chemotherapy,” “Nausea/Vomiting/Retching,” and “Cognitive/Behavioral/Diet/Exercise/Functional Exercise/Acupuncture/Moxibustion/Electroacupuncture/Acupressure/Auricular/Soothing/Music/Aromatic/Hypnotic/Imagination/Supplementation/Substitution/Non-pharmacological.” The English search words were “chemotherapy induced nausea vomiting/CINV/chemotherapy nausea/chemotherapy vomiting/chemotherapy emesis,” “cognitive/behav^∗^/diet/early mobilization/exercise^∗^/rehabilitation/transcutaneous electrical stimulation electrical stimulation/acupuncture/acupressure/herbal/moxibustion/auricular point/massage/relaxation/touch/systematic desensitization/music/aromatherapy/hypnosis/guided imagery/complementary/alternative/non-pharmacological,” and “prevention/treatment/therap^∗^/management/nurs^∗^/care/educat^∗^/train^∗^/Intervention.”

### 2.3. Inclusion and Exclusion Criteria

The inclusion criteria for literature were as follows: (i) the subjects were patients aged ≥ 18 years and underwent chemotherapy. (ii) The study was related to nonpharmacological prevention and treatment of CINV. (iii) The types of literature included clinical decisions, guidelines, evidence summaries, recommended practices, systematic reviews, and expert consensus. (iv) Publication language was Chinese or English.

Literature was excluded if (i) the researchers were unable to obtain the full text or an article was incomplete; (ii) it was published repeatedly; (iii) it was a conference report; (iv) it was of low quality.

### 2.4. Criteria for Literature Quality Evaluation

Evaluation criteria were selected for quality evaluation based on literature type. The quality evaluation criteria for guidelines were from the Appraisal of Guidelines for Research and Evaluation Instrument (AGREE II) [6]. Systematic reviews were evaluated using the JBI Systematic Review Quality Evaluation Tool (2016 Edition) [7]. The included expert consensus was evaluated using the JBI Expert Consensus Quality Evaluation Tool (2016 Edition) [8]. Clinical decisions, evidence summaries, and recommended practices were assessed by tracing the references and evaluating the quality according to the original study corresponding to the extracted evidence items.

### 2.5. Evidence Quality Evaluation

Two researchers independently completed the quality evaluation of literature. Conflicts of evaluation opinions between the researchers were resolved through discussion or through evaluation of the literature by a third investigator. When evidence conclusions from different sources were repeated or conflicting, researchers followed the inclusion principle of prioritizing evidence based, high quality, and more recently published evidence.

## 3. Results

### 3.1. General Characteristics of the Included Literature

A total of 2985 articles were retrieved for this study, with 2708 remaining after removing duplicates. 59 articles were obtained after primary screening. Finally, 22 articles were obtained after full-text reading and rescreening, including 3 clinical decisions, 2 guidelines, 1 evidence summary, 2 recommended practices, 13 systematic reviews, and 1 expert consensus (Table 1).

### 3.2. Quality Evaluation Results of the Included Studies

AGREE II was used to evaluate the quality of 2 guidelines [12, 13]. The results revealed the ranges of domain standardization shown in Table 2. The study designs of the 2 guidelines were complete and of high quality; all were approved for inclusion. Among the 13 included systematic reviews [17, 29], 6 were in English literature [17, 19, 21, 22, 24, 27], and 7 were in Chinese [18, 20, 23, 25, 26, 28, 29]. The methodological quality of the 13 systematic reviews is shown in Table 3. In general, all systematic reviews had relatively complete study designs and were overall of high quality. Thus, all were approved for inclusion. Only 1 expert consensus was included in this study [30]. The evaluation results of all items were “yes”; its study design was complete, and it was of high quality; therefore, it was approved for inclusion. In addition, the researchers conducted quality evaluations for the evidence summary and recommended practices by tracing references. They found that the overall quality of the evidence summary by Lingxue et al. [14] was excellent, and the 2 recommended practices by Gu and Li [15] and Lingli and Jing [16] were great, so all were approved for inclusion.

Q: question.

### 3.3. Evidence Summary and Description

All included evidence was graded using the Australian JBI Evidence-Based Health Care Centre Evidence Recommendation Rating System (2014 Edition) [31]. According to the validity, feasibility, suitability, and clinical significance of the evidence, the recommendation level of evidence was determined as grade A or grade B based on the JBI recommendation grading. Finally, 15 pieces of relevant evidence were extracted from included studies. These were then divided into 3 aspects: risk factor assessment, nursing process specification, and nonpharmacological interventions for CINV (Table 4).

## 4. Discussion

### 4.1. Scientific Validity of Evidence

The quality of the included studies directly affects the accuracy and reliability of evidence. In this study, we systematically searched the relevant databases, strictly screened and evaluated the quality of the literature, and extracted and integrated the best evidence from clinical decisions, guidelines, evidence summaries, recommended practices, systematic reviews, and expert consensus involving nonpharmacological management of CINV. The two included guidelines [12, 13] have rigorous formulation processes, reliable methodologies, and high overall quality. The included evidence summary [14] and two recommended practices [15, 16] were found to be of good quality based on tracing the references. All thirteen systematic reviews included were high-quality articles, five of those systematic reviews [18, 21, 26, 28, 29] met all criteria for excellent-quality research. The two researchers strictly followed the principles of rigor, transparency, science, and standardization and avoided the influence of subjective consciousness as much as possible. In the process of evidence screening, extraction, translation, and synthesis, the researchers compiled and presented the best current evidence in this field.

### 4.2. Clinical Utility of Evidence

#### 4.2.1. Risk Factor Assessment

Chemotherapy drug related factors and patient intrinsic factors can affect CINV [10]. Among them, the emetic potential of chemotherapeutic agents is the most important factor for CINV [12]. According to the risk of vomiting in patients without antiemetic prophylaxis, chemotherapeutic agents can be divided into four emetic risk levels: high (>90%), moderate (30-90%), low (10-30%), and mild (<10%) [9]. As examples, representative drugs with high emetic risk include cisplatin; drugs with moderate emetic risk include oxaliplatin; drugs with low emetic risk include docetaxel; drugs with mild emetic risk include asparaginase [30]. The emetic potential of drugs varies according to their type, dose, concentration, rate of administration, and route of administration [12]. Different chemotherapy regimens and whether they used cyclically will affect the CINV. For combination chemotherapy regimens, the level of emesis is determined by first identifying the agent with the strongest emetic potential in combination therapy and then assessing the relative contribution of other agents [9]. Clinically, the population most prone to CINV is female chemotherapy patients under 50 years of age, who consume more than 100 g alcohol per day and have poor activity levels, physical status, anxiety, and underlying diseases and a previous history of motion sickness, pregnancy vomiting, or CINV [12, 30]. Based on these risk factors, nursing staff should assess a patient's risk of CINV at the inception of the treatment process, perform preventive intervention, and address CINV based on the patient's personal characteristics and current chemotherapy regimen. At present, CINV has not been assessed as an independent symptom in clinical practice, and there is a dearth of standardized, unified tools for continuous assessment and monitoring of CINV. Clinical and scientific research tends to use patient self-stated CINV assessment tools [36]. MAT developed by the Multinational Association for Supportive Care in Cancer (MASCC) is most commonly used and recommended to assess CINV [10, 15]. The MAT instrument contains 8 items containing whether acute or delayed CINV occurs, duration, frequency, and self-vomiting experience score (0 to 10 points). The MAT tool's Cronbach's *α* values are 0.77 and 0.82 for patient self-assessment and primary caregiver assessment, respectively [37]. It is recommended that patients use MAT to evaluate their past 24-hour CINV from the 1st to 5th, 7th, 14th, and 21st days after chemotherapy; this helps medical staff observe and monitor emesis throughout the chemotherapy cycle [15].

#### 4.2.2. Nursing Process Specification

Jie and Chunlan [38] conducted a qualitative interview with nurses in the oncology department in a hospital in China and found that the medical staff were not adequately attentive to patients' CINV and lacked a systematic and standardized perception of how to manage it. It is recommended that nursing administrators develop individual and organizational action plans, including carrying out standardized training on CINV (causes, symptoms, types, evaluation tools, treatment principles, precautions of commonly used drugs, etc.) and establishing a whole-process management process for CINV (inpatient management and discharge follow-up management) [16]. To ensure CINV is properly measured and addressed, it is recommended that nursing staff provide health education on CINV related knowledge to the patients, teach the patients to self-assess the MAT score, and improve the health knowledge level of the patients on CINV during hospitalization. Place a vomiting record sheet at the bedside or foot of the bed to record the vomiting time, frequency, contents, and MAT score which can transparent the patients' nausea and vomiting information to promote the communication between doctors and nurses [15, 16]. The doctor can adjust the antiemetic program as needed based on the vomiting record sheet. Health staff followed up with chemotherapy patients via telephone or WeChat group after discharge from the hospital. The follow-up contents included the psychological impact of nausea and vomiting, time, frequency, amount, contents, and MAT scores [30]. With this information, medical staff could detect and intervene on delayed CINV in a timely manner [15].

#### 4.2.3. Nonpharmacological Interventions for CINV

Nonpharmacological interventions are often used as complementary therapies for CINV. Patients love these treatments because they are affordable, convenient, and easy to use. These interventions include auricular point therapy, aromatherapy, ginger therapy, music therapy, relaxation therapy, diet therapy, acupressure therapy, acupuncture therapy, and moxibustion therapy. It is recommended to use acupressure, acupuncture, and other comprehensive methods combined with antiemetics to relieve CINV symptoms in cancer patients, which effect is superior to traditional drug therapy alone [14].

*(1) Auricular Point Therapy*. Auricular point therapy can be used as an adjuvant therapy in the prevention and treatment of CINV [17]. This therapy regulates a patient's physical and psychological function through meridians and acupoints; it is noninvasive, has relatively few side effects, safe, effective, and affordable. Hong [39] used auricular point therapy to effectively relieve CINV symptoms and enhance quality of life in gastric cancer chemotherapy patients. In clinical practice, auricular point treatment is often selected using *Vaccinium vulgare* seeds or magnetic beads. The Shen men, stomach, sympathetic, or subcortical are often selected as the main points. The liver and spleen are often selected as matching points. A compression effect is then created from the De-qi acuesthesia [17, 18, 32]. Pan et al. [32] combined antiemetic drugs with auricular point sticking to patients with platinum-based chemotherapy from the 1st to 5th day of their chemotherapy cycles, pressed 3 times a day for 2 minutes on each point on both ears. This proved the efficacy of auricular points for treating CINV. Based on findings from the above-mentioned main acupoints, Yufei et al. [40] added lung acupoints for patients of Qi and Yin deficiencies, kidney acupoints for kidney-Yang deficiency, and heart acupoints for Qi stagnation and blood stasis, which indicate the positive effect of additional acupoints in the treatment of CINV after traditional Chinese medicine syndrome differentiation.

*(2) Aromatherapy*. Aromatherapy is a therapy using essences, essential oils, and aromatic hydrosols extracted from aromatic plants [19]. Aromatherapy is considered to be an economic effective complementary treatment for CINV because it can relieve gastrointestinal discomfort symptoms by Qi. Aromatherapy has antiemetic effects on acute and delayed CINV. Rosemary, bergamot, peppermint, ginger, and lemon are all common aromatic plants. Aromatic preparations with different extract concentrations and preparation conditions have different efficacy in treating CINV [41]. It is recommended to use essential oils containing peppermint, which has the strongest antiemetic effect. Some patients may have intolerance to single essential oils. In this case, compound essential oils are more suitable [20]. Moreover, inhaled aromatherapy is superior to other interventional approaches such as smear or oral administration [19]. The theoretical basis of inhalation aromatherapy is that aromatic substances act on nasal olfactory cells after absorption through the nasal meatus to produce biological signals and promote the release of antiemetic-related neurotransmitters from the brain, thereby preventing and treating CINV. Inhaled aromatherapy is more user-friendly than other methods, avoids direct contact between essential oils and human skin, and reduces the occurrence of potentially dangerous events such as dermatitis and allergic reactions.

*(3) Ginger Therapy*. UpToDate summarized six randomized controlled trials exploring the effect of ginger as an adjuvant therapy for CINV. The results are inconsistent. Three studies found ginger to be beneficial to this end, while the remaining three failed to find any benefits [9]. Patients with different constitutions have different tolerance to ginger. Some patients experience aggravated nausea and vomiting after using ginger [11]. At this moment, there is insufficient evidence available to draw a firm conclusion about using ginger therapy to prevent and manage CINV; thus, it should be used cautiously [9, 11, 21].

*(4) Music Therapy*. Music therapy is based on psychology. It entails applying a unique acoustic frequency to regulate physical and psychological symptoms, which is conducive to divert patients' attention, stabilizing mood, and ultimately reducing CINV [22]. Clinical research mostly focuses on the music type, intervention frequency, etc. Yixiao and Caixia [23], after comprehensively evaluating patients' age, music preferences, cultural backgrounds, and beliefs, constructed a music library for CINV patients. This effectively reduced CINV severity, suggesting that music therapy has a positive effect on CINV. Nursing staff should choose appropriate, soothing music based on patients' hobbies, lifestyles, habits, living environments, and other personalized characteristics. It is recommended to play the selected music for 30-60 minutes [23, 42].

*(5) Relaxation Therapy*. Relaxation therapy mostly refers to progressive muscle relaxation—the gradual contraction and relaxation of muscle groups to relax the whole body, thereby reducing the excitability of the sympathetic nervous system and the sensitivity of the emesis center, which has a positive effect on the treatment of CINV [24, 30]. To this end, the Chinese Medical Association Audio and Video Publishing House published a progressive muscle relaxation training course. Dan et al. [33] applied this tutorial to guide and assist patients' relaxation training before chemotherapy, after chemotherapy, and after discharge from hospital, 25 minutes each time once per day for a total of 7 days. The research showed that relaxation therapy could effectively relieve the CINV symptoms and fatigue of lung cancer patients. This course is low cost and easy to learn; in addition, it is not limited by time or region and is worthy of clinical application [33].

*(6) Diet Therapy*. Diet therapy for CINV primarily includes implementing reasonable eating habits, eating patterns, and food choices. Nurses should instruct CINV patients to avoid greasy, spicy, sweet, and irritating foods with strong odors. Patients should choose foods that are high calorie, high protein, low fat, and vitamin rich. Ideally, patients should consume digestible liquid or semiliquid diets as much as possible [12]. Patients should regulate their eating patterns, and it is advisable to eat small meals at frequent intervals and drink a small amount of water several times per day. Studies have found that refusal to eat is not conducive to CINV symptom management and can lead to a nearly sevenfold increase in CINV incidence [43]. Patients can try to eat popsicles, lemon slices, and menthol or chew gum to remove mouth odors and improve their appetite by improving the taste of food [12, 25, 30]. Moreover, Li et al. [34] formulated a phased diet table and individualized recipes for gynecological cancer patients before, during, and after various chemotherapy stages according to disease characteristics, diagnosis, treatment regimen, and dietary nutrients. This provides a reference for the clinical practice of nutritional support and dietary management of chemotherapy.

*(7) Acupressure Therapy*. The goal of acupressure is to dredge the meridians, invigorating the spleen and stomach by stimulating local or systemic acupoints. Neiguan is the most commonly used acupoint in clinical practice [26]. Pressing it can effectively decrease the severity of CINV in cancer patients and reduce the incidence of vomiting, retching, and nausea [26]. It is advised that medical staff perform Neiguan (bilateral) acupressure on chemotherapy patients once per day from the first day of chemotherapy until the end of the chemotherapy cycle [13]. In addition, it is recommended that Neiguan point be used in combination with Hegu and Zusanli acupoints, as this facilitates the exertion of synergistic effects and enhances antiemetic efficacy [14]. Acupressure can decrease the degree of acute and delayed CINV. Self-acupressure therapy has a positive impact on CINV as well. All in all, it is recommended that medical staff perform acupressure therapy on patients based on professional judgment and patient preference [14].

*(8) Acupuncture Therapy*. As a characteristic method of external treatment in traditional Chinese medicine, acupuncture therapy regulates the body's neurohumoral system along the meridians, which affect gastrointestinal motility and visceral sensations, thereby treating CINV [9, 11]. The Zusanli, Neiguan, Zhongwan, Tianshu, and Gongsun points are common acupoints in clinical practices [29]. Tai [44] applied descending inverse group point acupuncture therapy combined with conventional antiemetic drugs 1 to 5 days after chemotherapy and found that it decreased the severity of CINV and had a significantly better effect on CINV than drug therapy alone. It is recommended to treat patients with filiform needle acupuncture on the Neiguan (bilateral) point 2 hours before the first day of chemotherapy, and then perform it once per day for a total of seven days [13]. Alternatively, some patients were treated with transcutaneous electrical acupoint stimulation at Neiguan (bilateral) or Yongquan (bilateral) points twice a day for 30 minutes from the first to third day of the chemotherapy cycle [13].

*(9) Moxibustion Therapy*. Moxibustion passes through the venation to reach the meridians and plays a role in regulating Qi, invigorating spleen, and stopping vomiting in the stomach. Modern studies [45] have found that moxibustion improves gastrointestinal function by reducing gastric mucosal injury and increasing serum gastrin and motilin levels. Moxibustion, combined with antiemetic drugs, is effective in reducing CINV severity and frequency [11, 27]. Medical staff can select from moxa stick moxibustion, moxa cone moxibustion, and partitioned moxibustion or other moxibustion approaches; take rotary moxibustion, sparrow pecking moxibustion, and round-trip moxibustion or other different manipulation techniques. Zusanli, Zhongwan, Shenque, and Neiguan are common acupoints in clinical practices [35]. Qian et al. [46] found that the effect of moxibustion on CINV is almost congruent on different treatment phase, but patients are more satisfied and acceptable with this treatment during intermittent chemotherapy periods, which provides a reference for clinical medical staff to choose an appropriate time to apply this intervention for CINV.

## 5. Conclusions

This study systematically summarizes the best evidence for nonpharmacological management of CINV from three standpoints: risk factor assessment, nursing process specifications, and nonpharmacological interventions for CINV. Overall, evidence on this topic was of great quality and has scientific validity and clinical utility. In this study, nonpharmacological interventions such as auricular point therapy and aromatherapy are the most ubiquitous techniques for traditional Chinese medicine nursing in clinical practice, which can treat CINV effectively. However, the frequency and duration of some interventions have not yet been established or standardized; clinical operability and generalizability remain insufficient. Future studies should be of higher quality, have larger sample sizes, be conducted in multicenter settings, establish a unified theoretical system, and practice norms to provide more scientific and standardized guidance about nonpharmacological interventions for CINV. Medical staff need to comprehensively evaluate the feasibility of nonpharmacological interventions, fully consider patients' wishes, follow the principle of individualization, and prudently apply evidence to clinical practice. Health staff should specifically analyze the obstacles and facilitating factors in evidence application, formulate targeted action strategies, implement changes at the individual and organizational levels, and implant high-quality evidence in clinical practice.

## Figures and Tables

**Table 1:** Characteristics of included studies (*n* = 22).

Author	Evidence type	Time	Literature theme	Source
Peishi et al. [9]	Clinical decision	2021	Prevention and treatment of CINV in adults	UpToDate
Shadan et al. [10]	Clinical decision	2021	Pathophysiology and prediction of CINV	UpToDate
William et al. [11]	Clinical decision	2018	Risk of vomiting in the toxicity of chemotherapeutic agents and alternative therapies	DynaMed
Shiying et al. [12]	Guideline	2014	Guideline for prevention and treatment of vomiting associated with cancer therapy	Medlive
Jingwen et al. [13]	Guideline	2021	Guideline for acupuncture and moxibustion prevention and treatment of nausea after chemotherapy	Wanfang
Lingxue et al. [14]	Evidence summary	2020	Summary of best evidence for acupressure relieving CINV in cancer patients	Cnki
Gu and Li [15]	Recommended practice	2016	A best practice implementation project of assessment and management of CINV	JBI
Lingli and Jing [16]	Recommended practice	2016	Recommended practice for evaluation and management of CINV	Cnki
Chen et al. [17]	Systematic review	2021	Efficacy of auricular acupressure in prevention and treatment of CINV	ProQuest
Shaomei [18]	Systematic review	2015	Auricular point sticking therapy for CINV	Wanfang
Beloni et al. [19]	Systematic review	2021	Effectiveness of inhaled aromatherapy on CINV	Ovid
Pengcheng et al. [20]	Systematic review	2020	Aromatherapy for prevention of CINV	Wanfang
Crichton et al. [21]	Systematic review	2019	Efficacy of ginger in ameliorating CINV	PubMed
Wei et al. [22]	Systematic review	2020	Music interventions for CINV	Ovid
Yixiao and Caixia [23]	Systematic review	2020	Individualized music intervention for CINV	Wanfang
Xu et al. [24]	Systematic review	2020	Progressive muscle relaxation in preventing and alleviating of CINV	ProQuest
Tingyu et al. [25]	Systematic review	2016	Effect of diet nursing of CINV	Cnki
Feng et al. [26]	Systematic review	2020	Neiguan acupressure improving CINV effect	Wanfang
Huang et al. [27]	Systematic review	2017	Moxibustion for CINV	WOS
Ziyan et al. [28]	Systematic review	2021	Moxibustion in preventing and treating CINV	Wanfang
Shan [29]	Systematic review	2020	Acupuncture therapy for CINV	Cnki
Wenqi et al. [30]	Expert consensus	2019	Chinese expert consensus on prevention and treatment of CINV	Wanfang

**Table 2:** Methodological quality evaluation of the guidelines.

Included guideline	Percentage of standardization in each domain of the guide (%)						≥60% number of fields (number)	≥30% number of fields (number)	Recommended level
	Scope and purpose	Stakeholder involvement	Rigor of development	Clarity of presentation	Clarity of presentation	Editorial independence			
Shiying et al. [12]	75.0	44.4	41.7	45.8	31.7	16.7	1	5	B
Jingwen et al. [13]	72.2	69.4	60.4	45.8	46.7	12.5	3	5	B

**Table 3:** Methodological quality evaluation of systematic reviews.

Author	Q1	Q2	Q3	Q4	Q5	Q6	Q7	Q8	Q9	Q10	Q11	Q12
Chen et al. [17]	Yes	Yes	Yes	Yes	Yes	Yes	Yes	Yes	No	Yes	Yes	Yes
Shaomei [18]	Yes	Yes	Yes	Yes	Yes	Yes	Yes	Yes	Yes	Yes	Yes	Yes
Beloni et al. [19]	Yes	Yes	Yes	Yes	Yes	Yes	Yes	Yes	No	Yes	No	Yes
Pengcheng et al. [20]	Yes	Yes	Yes	Yes	Yes	Yes	Yes	Yes	No	Yes	Yes	Yes
Crichton et al. [21]	Yes	Yes	Yes	Yes	Yes	Yes	Yes	Yes	Yes	Yes	Yes	Yes
Wei et al. [22]	Yes	Yes	Yes	Yes	Yes	Yes	Yes	Yes	Yes	Yes	No	Yes
Yixiao et al. [23]	Yes	Yes	Yes	Yes	Yes	Yes	Yes	Yes	Yes	Yes	No	Yes
Xu et al. [24]	Yes	Yes	Yes	Yes	Yes	Yes	Yes	Yes	No	Yes	Yes	Yes
Tingyu et al. [25]	Yes	Yes	Yes	Yes	Yes	Yes	Yes	Yes	No	Yes	No	Yes
Feng et al. [26]	Yes	Yes	Yes	Yes	Yes	Yes	Yes	Yes	Yes	Yes	Yes	Yes
Huang et al. [27]	Yes	Yes	Yes	Yes	Yes	Yes	Yes	Yes	No	Yes	Yes	Yes
Ziyan et al. [28]	Yes	Yes	Yes	Yes	Yes	Yes	Yes	Yes	Yes	Yes	Yes	Yes
Shan [29]	Yes	Yes	Yes	Yes	Yes	Yes	Yes	Yes	Yes	Yes	Yes	Yes

**Table 4:** Evidence summary of nonpharmacologic management for chemotherapy-induced nausea and vomiting (CINV).

Aspects	Evidence item		Evidence level	Recommendation level
Risk factor assessment	(1) Common risk factors for CINV are divided into chemotherapy drug related factors (emetic potential, dose, concentration, speed, mode of administration, concomitant use, and periodic use) and patient-intrinsic factors (gender, age, alcohol intake, anxiety, activity level, physical status, underlying diseases, previous history of motion sickness, pregnancy vomiting, or CINV) [10–12, 30].		1a	A
	(2) At each chemotherapy cycle, health care providers are invited to use MASCC assessment tool (MAT) developed by the Multinational Association for Supportive Care in Cancer for risk assessment [10]. From the 1st to 5th, 7th, 14th, and 21st days after chemotherapy, the patients used the MAT to self-evaluate their nausea and vomiting in the past 24 hours [15].		3c	B

Nursing process specification	(3) Through standardized training, nursing staff systematically learn the relevant guidelines and expert consensus of CINV and master the causes, symptoms, types, assessment tools, treatment principles, and precautions of commonly used drugs for CINV [16].		2d	A
	(4) During hospitalization, nursing staff provide patients with health education on CINV to administer the chemotherapy drugs on time, accurately; evaluate CINV and chemotherapy efficacy in time to obtain feedback that will help adjust the next cycle of a chemotherapy regimen. Place a vomiting record sheet at the bedside or foot of the bed to record vomiting time, frequency, contents, and MAT score can provide metrics on a patient's nausea and vomiting. This promotes communication between doctors and nurses [15, 16].		2d	B
	(5) After patients are discharged from the hospital, they were followed up via telephone or WeChat group. The follow-up contents include the psychological impact of nausea and vomiting, the time, frequency, amount and contents, and self-score using the MAT [30]. Nurses should detect and intervene delayed CINV in a timely manner [15].		5b	B

Nonpharmacological interventions for CINV	Auricular point therapy	(6) Auricular point treatment is often selected using Vaccinium vulgare seeds or magnetic beads. The Shen men, stomach, sympathetic, or subcortical are often selected as the main points. The liver and spleen are often selected as matching points. A compression effect is then created from the De-qi acuesthesia [17, 18, 32].	1a	B
	Aromatherapy	(7) Aromatherapy has antiemetic effects on acute and delayed CINV. It is best to use essential oils containing peppermint, which has a strong antiemetic effect. Some patients may have intolerances to single essential oils. Compound essential oils are more suitable when this is the case [20]. Moreover, inhaled aromatherapy is superior to other interventional approaches such as smear or oral administration [19].	1a	B
	Ginger therapy	(8) There is insufficient available evidence to recommend or oppose ginger therapy to prevent and manage CINV; ginger should be used cautiously [9, 11, 21].	1a	B
	Music therapy	(9) Music therapy is helpful for treating CINV [22, 23]. Nurse staff should choose appropriate soothing music based on patients' hobbies, lifestyles, habits, living environments, or other personalized characteristics. It recommends that music plays for 30 min-60 min [23].	1a	B
	Relaxation therapy	(10) Progressive muscle relaxation has positive effects on CINV [24, 30]. The progressive muscle relaxation training course published by the Chinese Medical Association audio and video publishing house is available regardless of time zone and region, and is worthy of clinical application [33].	1a	B
	Diet therapy	(11) Reasonable eating habits, eating patterns, and food choices can effectively prevent and treat CINV. Medical staff should formulate staged diet tables and individualized recipes for CINV patients according to disease characteristics, diagnosis and treatment plans, and dietary nutrients [12, 25, 30, 34].	1b	B
	Acupressure therapy	(12) Acupressure reduces the degree of acute and delayed CINV, and self-acupressure therapy has a positive effect on CINV. Neiguan acupoint is the most commonly used acupoint in clinical practice [26]. As soon as chemotherapy begins, medical staff can perform Neiguan (bilateral) acupressure on chemotherapy patients once per day until the end of the chemotherapy cycle [13].It is recommended that Neiguan point be used in combination with Hegu and Zusanli acupoints [14].	1a	A
	Acupuncture therapy	(13) Acupuncture therapy is effective in treating CINV [9, 11]. Common acupoints used in clinical practice are Zusanli, Neiguan, Zhongwan, Tianshu, and Gongsun points [29].It is recommended to treat patients with filiform needle acupuncture on the Neiguan (bilateral) acupoints 2 hours before the first day of chemotherapy, and then perform acupuncture once a day for a total of seven days [13]. Alternatively, patients were treated with transcutaneous electrical acupoint stimulation at Neiguan (bilateral) or Yongquan (bilateral) points twice a day for 30 minutes from the first to third day of each chemotherapy cycle [13].	1b	A
	Moxibustion therapy	(14) Moxibustion combined with antiemetic drugs is effective in decreasing the severity and frequency of CINV [11, 27]. Medical staff can select from moxa stick moxibustion, moxa cone moxibustion, and partitioned moxibustion or other moxibustion approaches; take rotary moxibustion, sparrow pecking moxibustion, and round-trip moxibustion or other different manipulation techniques. These are compatible with Zusanli, Zhongwan, Shenque, Neiguan, and other acupoints and can improve CINV status [35].	1a	B
	Comprehensive therapy	(15) It is recommended to combine acupressure, acupuncture, and other comprehensive methods with antiemetics to relieve CINV symptoms in cancer patients together. The effect is superior to traditional drug therapy alone [14].	5b	B

## Data Availability

The data during the current study are available from the corresponding author upon reasonable request.
